# Pharmacy Services beyond the Basics: A Qualitative Study to Explore Perspectives of Pharmacists towards Basic and Enhanced Pharmacy Services in Pakistan

**DOI:** 10.3390/ijerph17072379

**Published:** 2020-03-31

**Authors:** Muhammad Atif, Wajiha Razzaq, Irem Mushtaq, Iram Malik, Madiha Razzaq, Shane Scahill, Zaheer-Ud-Din Babar

**Affiliations:** 1 Department of Pharmacy, The Islamia University of Bahawalpur, Bahawalpur 63100, Pakistan; wajiharazzaq179@gmail.com (W.R.); iramalik686@gmail.com (I.M.); madiharazzaq25@gmail.com (M.R.); 2 Department of Education, The Islamia University of Bahawalpur, Bahawalpur 63100, Pakistan; irem_atif@yahoo.com; 3 School of Pharmacy, University of Auckland, Auckland 1142, New Zealand; s.scahill@auckland.ac.nz; 4 Department of Pharmacy, University of Huddersfield, Huddersfield HD1 3DH, UK; z.babar@hud.ac.uk

**Keywords:** Drug Regulatory Authority of Pakistan (DRAP), pharmacy services, pharmaceutical policy

## Abstract

Enhanced pharmacy services have been identified as a mechanism to address medicines and drug-related problems. The aim of the study was to explore the perspectives of practicing pharmacists on the scope of pharmacy service provision in Pakistan. This qualitative study was conducted at the Department of Pharmacy, the Islamia University of Bahawalpur (IUB). Face-to-face, in-depth interviews were conducted with practicing pharmacists at the university who were undertaking postgraduate studies. All interviews were audio-recorded, transcribed verbatim, and analyzed using thematic analysis. A total of 13 pharmacists were interviewed. The analysis of data yielded four themes and 12 subthemes. The themes included the current scenario of pharmacy services, the benefits of pharmacy services, barriers to implementation of pharmacy services, and strategies to improve their delivery. Pharmacist participants reported that patient-oriented pharmacy services have not been properly implemented in Pakistan. Pharmacists appear to be undertaking only conventional roles at various levels within the healthcare system. The participants indicated multiple benefits of patient-oriented pharmacy services, including safe and effective use of medicines, minimization of drug-related problems, and financial benefits to the healthcare system. Based on the findings, policy-makers are required to take the necessary steps to overcome pharmacist-related and policy-related barriers associated with the implementation of patient-oriented pharmacy services in Pakistan.

## 1. Background

Over recent decades, there has been considerable worldwide increase in the morbidity and mortality associated with chronic diseases. This has been linked with lack of individualized patient care and consequent medication-related issues [1]. To this end, the World Health Organization (WHO) and the International Pharmaceutical Federation (FIP) have recognized the need to develop pharmacy services as a way to meet the fast-growing demand for safe and quality uses of medicines, alongside affordable healthcare service provision [2]. The European Society of Pharmacy and the Canadian Pharmacist Association have also stressed the need to expand the clinical services provided by pharmacists in both hospital and community settings [3,4]. As a result, the role of the pharmacist has shifted from product-oriented services to patient-oriented services [5], and advanced pharmacy services are progressively becoming vital within developed healthcare systems [6]. Nevertheless, despite a significant body of international research reporting positive health and economic outcomes associated with the implementation of enhanced cognitive pharmacy services [7,8,9], there have been barriers to successful implementation in developing countries.

Pakistan is a low and middle income country (LMIC) that has been recently ranked lower than all other South Asian countries at 152th out of 189 countries, with a Human Development Index (HDI) value of 0.560 [10]. Over the past couple of decades, the Pakistani healthcare system has suffered a great deal from the burden of communicable and non-communicable diseases, limited healthcare workforce, reduced health sector investment, and a lack of health insurance schemes [11,12]. Pakistan is struggling with a number of threats to the optimal use of medicines, including high prevalence of medication errors (causing deaths of 0.5 million people annually [13]) and adverse drug reactions (detected in 60% adults and 40% children [14]), misuse of controlled substances (by 6.7 million individuals [15]), and excessive self-medication [16]. There is also a growing list of medicine-related issues that demand immediate participation of pharmacists in the patient’s welfare, including over-the-counter (OTC) availability of prescription medicines, inappropriate use of medicines, formulation issues, unsafe storage and disposal of medicines, and poor availability of medicines [16,17,18,19,20,21]. Additionally, poor health in the female population, elderly, and medically underserved rural populations in Pakistan need scalable and affordable community pharmacy services, such as health screening (e.g., diabetes, cholesterol, osteoporosis), timely immunizations, pain control, and home-based care delivery and family planning services [22,23].

In Pakistan, the three year Bachelor of Pharmacy degree was first initiated in 1948 [24]. However, the regulations and job description for the pharmacy profession were defined decades later in the Pharmacy Act, 1967 [24]. The pharmacy degree has seen a transition from the four year B.Pharm program in 1979 to the five year Pharm-D program in 2004 [24]. Along with the improvements in the pharmacy curriculum, the goal of implementing enhanced cognitive pharmacy services and the recruitment of pharmacists in hospitals was also called for in Pakistan’s National Drug Policy 2003 [20,24]. Moreover, in recognition of the importance of delivering pharmacy services, the Federal Government of Pakistan introduced the Drug Regulatory Authority of Pakistan (DRAP) Act 2012 to provide the legislative impetus to develop high quality pharmacy services throughout the country [25]. According to the DRAP Act 2012 pharmacy services involve, “services offered by the pharmacists ranging from existing basic services (i.e., dispensing and counselling, procurement, storage, distribution and management of therapeutic goods) to enhanced pharmacy services (i.e., prescription monitoring and drug utilization review (DUR), pharmaceutical care, pharmacovigilance, pharmaco-economics, services at drug information centers and poison control centers) at each level, such as pharmacy, clinical, hospital, and community levels” [25]. Later in 2014, there was another worthwhile step towards strengthening the expertise of the pharmacist in securing the role as a clinical service provider—the introduction of the Department of Pharmacy Practice in the private and public sector universities [26].

Despite these ongoing government-level efforts, as well as literature and policy calling for the implementation of pharmacy services, lack of recognition of the full scope of pharmacy services has remained a significant impediment for pharmacists to gain a foothold as competent and recognized healthcare professionals in Pakistan [24]. With very few exceptions, advanced patient-oriented pharmacy services are not offered at hospitals or in the private sector drug retail outlets (community pharmacies) in Pakistan [19,20,27]. Barriers to the recognition and expansion of pharmacy services in both hospital and community settings include suboptimal enforcement of rules and regulations, administrative barriers, inadequate number of pharmacy workers (only 0.06 pharmacists available for about 10,000 people—6/1,000,000), lack of academic capability of the pharmacist in terms of patient-oriented clinical services, lack of collaboration with other healthcare professionals, and lack of awareness regarding the existence and role of the pharmacist amongst the Pakistani public [20,24].

Given the evidence that the recognition and implementation of advanced pharmacy services have helped address the void in public health across many developed nations [9], it is difficult to reconcile why patient-oriented roles of pharmacists remain untapped and are poorly recognized in Pakistan. In this regard, the perspective of pharmacists (who are key stakeholders in the healthcare sector) is important to understand in order to fill the literature gap and gain a comprehensive understanding of the current pharmacy sector landscape and the emerging challenges associated with the implementation of pharmacy services. Therefore, this study was conducted to explore the perspectives of pharmacists about the scope of pharmacy service provision in Pakistan.

## 2. Method

### 2.1. Study Setting

The study was conducted at the Department of Pharmacy, the Islamia University of Bahawalpur (IUB). The IUB is located in the district of Bahawalpur, the Punjab province of Pakistan. The University was established in 1925 and encompasses 45 departments and 74 disciplines. Courses are available in Engineering, Computer Science, Arts and Humanities, Life Sciences, Sports Science, and Pharmacy [28]. In 1990, the Department of Pharmacy was established at the Khawaja Fareed Campus, Bahawalpur. At the time of this study, approximately 200 postgraduate students were enrolled at the Department of Pharmacy, IUB.

### 2.2. Study Participants

Postgraduate students with part-time employment in the private sector drug retail outlets (community pharmacies) or public sector hospital pharmacies and who were on study leave for their postgraduate education were invited to participate in the study. To assure the reliability and competence of the informants, and ultimately the quality of the data gathered, participants with a minimum work experience of five years duration were eligible to participate in the study.

### 2.3. Study Design

In-depth, semi-structured, face-to-face interviews were used as part of a qualitative study design to explore pharmacists’ views on the scope of pharmacy services in Pakistan. The qualitative method strives to understand phenomena in depth, where little information has previously been generated and for which the aim is not to generalize [29].

### 2.4. Interview Schema Development

The interview schema was developed through an extensive literature search [30,31,32] and with the aim of answering the research questions. The schema was developed through regular meetings amongst the research team and is outlined in Supplementary File S1. Before conducting the interviews, the schema was pilot tested with two pharmacists in order to confirm that the questions were understood as posed. This procedure took about 35 min for each interview. As a result of these pilot interviews, meaningful changes were made to the schema. The interview data from the pilot process were not included in the final analysis. Piloting of the interview schema involved repetitive subtle modifications during the study to ensure that emergent themes were accounted for in the ongoing interviews. The term pharmacy service in the schema is related to the definition of services under the DRAP Act 2012.

### 2.5. Data Collection

The data were collected from 1st May 2019 to 31st July 2019. Saturation point criteria were used to determine the point at which the sample size was deemed adequate. The study participants were selected through a two-stage process, involving purposive and convenience sampling techniques [33]. In the first step, identified participants were purposively approached at either their work place or at the university during working hours (8 am to 4 pm) and eligible participants were invited to participate in the study. In the second step, the study participants who consented to participate in the study were selected and interviewed at a time and place that was agreeable to both parties. All interviews were conducted in Urdu (native language of Pakistan). The semi-structured interviews were conducted by Wajiha Razzaq. All interviews were audio recorded and transcribed verbatim. The study participants were given the opportunity to listen to the recorded interviews and read the written transcripts.

### 2.6. Data Analysis

The data were analyzed using the thematic analytic approach of Braun and Clarke [34]. Six phases of data analysis make up their thematic approach, i.e., familiarization with the data, generation of initial codes, searching for themes, reviewing themes, defining and naming themes, and producing the report [34]. Data familiarization started at an initial phase when audio recordings were carefully listened to several times, transcribed verbatim, and then translated into English from Urdu. For the validation of the translation procedure, a forward and backward translation method was tested on a group of transcripts [34] and was found to be dependable [29]. Open coding was undertaken to generate the initial codes that addressed the study objectives. The coded information was reduced and ordered as themes and subthemes emerged. The final themes were reviewed by all authors. Cross-checking of the emergent themes and conclusion of the study was done via regular meetings of the authors. In the case of non-consensus, the final decision was made by the senior author(s).

### 2.7. Ethics Approval

Ethical approval was obtained from the Pharmacy Research Ethics Committee (PREC) at the Islamia University Bahawalpur (Reference: 69/S-2019-/PREC, dated 02 May 2019). Written informed consent was obtained from the study participants after explaining the aims and objectives of the study. The identities of the study participants remain confidential by assigning identification codes to them. A pressure-free and trustworthy environment was maintained throughout the interview. The participants were given the freedom to raise concerns, skip any question, or even withdraw from the interview at any time during the study without giving reasons, although none chose to quit the interview and all the raised concerns were candidly answered.

### 2.8. Notes

In this study, the phrase “renting out or leasing out license of pharmacist” refers to “a contract between two parties, the lessor (owner of asset) and the leasee (user), in which a non-qualified medical store/pharmacy owner (leasee) pays a signed amount to the pharmacist (lessor having a pharmacy degree and license) for the use of rights granted under that license.”

## 3. Results

A total of 13 pharmacists were interviewed. The average interview duration was 26.4 min (range 22 to 30 min, SD = 2.5). The mean age of the study participants was 36 years (range 32 to 42 years, SD = 3.4). The characteristics of the respondents are outlined in Table 1.

The analysis of data yielded four themes and 12 subthemes. The themes included current scenario of pharmacy services, benefits of pharmacy services, strategies to improve pharmacy services, and barriers to the implementation of pharmacy services.

### 3.1. Theme 1: Current Scenario of Pharmacy Services

All respondents were familiar with the term “pharmacy services”. According to respondents, these are the services offered by pharmacists directed at patient care. The majority of participants had an understanding of the terms dispensing, counselling, distribution, prescription monitoring, storage, procurement, and pharmacovigilance (12 out of 13 respondents). However, most of the pharmacists were unaware that domains such as pharmaco-economics (8 out of 13 respondents), posology (11 out of 13 respondents), and pharmaco-epidemiology (7 out of 13 respondents) were also part of sanctioned pharmacy services according to the DRAP Act 2012.

Most of the pharmacists (10 out of 13 respondents) were of the opinion that foundation services, such as dispensing, storage, procurement, and sale of medicines, are currently being offered by pharmacists in Pakistan. Almost 70% of pharmacist respondents (9 out of 13 respondents) reported that counselling is of low quality and was being performed properly at a few private hospitals but not government hospitals. The same proportion of pharmacists (9 out of 13 respondents) were of the opinion that prescription monitoring, pharmacovigilance, poison control centers, posology, selection of medicines, drug utilization review, and evaluation were being partially offered in healthcare institutes. Eight out of 13 respondents stated that drug use studies and pharmaco-economics were uncommon practices in healthcare settings in Pakistan. As an overview, the theme related to the current scenario of the pharmacy services being offered in Pakistan, along with subthemes, categories, and exemplar quotations, is provided in Table 2.

### 3.2. Theme 2: Benefits of Pharmacy Services

Numerous advantages for the provision of pharmacy services were indicated by participants. Better quality of life, improved adherence to therapy, and reduced drug-related problems (DRPs) were the patient-related benefits that emerged in discussion about the implementation of pharmacy services in Pakistan. Similarly, reduced burden on healthcare professionals and financial gains were the healthcare-system-related benefits explained by the study participants. As an overview, the theme related to benefits of implementing pharmacy services in Pakistan, along with categories and exemplar quotations, is provided in Table 3.

### 3.3. Theme 3: Barriers in Implementation of Pharmacy Services

The study participants indicated that having a limited number of pharmacists, inadequate number and type of roles and training opportunities for pharmacists (8 out of 13 respondents), lack of patient knowledge about pharmacists and their role in healthcare delivery (6 out of 13 respondents), and suboptimal recognition by the government (8 out of 13 respondents) of pharmacists were the major barriers hindering the implementation of pharmacy services at all healthcare levels in Pakistan. Moreover, renting out pharmacist licenses (8 out of 13 respondents) and negative perceptions from doctors about pharmacists (7 out of 13 respondents) were the other barriers to successful implementation of pharmacy services at community and hospital healthcare levels, respectively. The theme related to barriers in implementation of pharmacy services, along with subthemes, categories, and exemplar quotations, is presented in Table 4. 

### 3.4. Theme 4: Strategies to Improve Pharmacy Services

The participants elaborated on various strategies for the improvement of pharmacy service provision in Pakistan. Most of the study participants emphasized the need to provide training opportunities for pharmacists (11 out of 13 respondents). Just under half (n = 6) of the pharmacists stated that pharmacy services could be improved by educating patients about the benefits of these services and by promoting the role of pharmacists in the healthcare sector, especially in community settings. At the hospital level, better interactions between doctors and pharmacists was deemed crucial by the study participants to improve the delivery of pharmacy services. As an overview, the theme related to strategies to improve pharmacy services in Pakistan, along with subthemes, categories, and exemplar quotations, is presented in Table 5. 

## 4. Discussion

In Pakistan, pharmacists have added value to the healthcare system as either manufacturers, purveyors, and dispensers of medicinal products, or as clinical service providers [35]. Notwithstanding this, the dominance within healthcare personnel lies with the physicians and nurses. Tacit denial of pharmacists’ expertise and importance is evident in the recent Health Economic Survey 2018–2019, in which pharmacists were not listed as working healthcare professionals [36]. However, due to the increasing complexity of chronic disease and associated medicine use, there is a growing need for enhanced pharmacy services in hospital and community settings. Little is known about how pharmacists in Pakistan perceive this situation and an in-depth qualitative analysis was implemented with a view to better understanding the situation, whilst gaining the attention of health policy-makers about these unrecognized pharmacy services. This study yielded four themes and describes the perspectives of pharmacists regarding the scope of implementation of pharmacy services in Pakistan, thereby extracting information about the current scenario, benefits of pharmacy services, challenges in their implementation, and strategies to better leverage enhanced pharmacy service provision.

The emergent findings suggest that most of the pharmacist participants were familiar with the concept of “pharmacy services”. Most of the respondents also had a sound understanding of traditional pharmacy services (i.e., dispensing, counselling, distribution, storage, and procurement). Similar to this study, the findings of studies from Middle-Eastern countries, such as Jordan, Iran, and Lebanon, indicate that most of the participants understand the concept of basic pharmacy services [37,38]. As far as newly emerging enhanced pharmacy services are concerned, the participants in our study demonstrated limited perceived knowledge about pharmaco-epidemiology, posology, and pharmaco-economics, with the exception of pharmacovigilance. In contrast, studies from developed countries have reported that most pharmacists were well-equipped and had comprehensive knowledge about their role in pharmacovigilance, pharmaco-epidemiology, posology, and pharmaco-economics, owing to well-established guidelines and vigilant regulatory bodies [3,7,39]. Taking this into account, better knowledge about pharmacovigilance among pharmacists in Pakistan is likely to be attributable to the recent implementation of a National Pharmacovigilance Center [40].

When asked about the services currently being provided in healthcare settings, the participants in this study viewed pharmacy services to be in their infancy in Pakistan. They further elaborated that conventional pharmacy services, such as dispensing, storage, sale, procurement, and distribution, were offered at the government, private, and community healthcare levels, whereas counselling, posology, selection of medicines, prescription monitoring, pharmacovigilance, and drug utilization review were partially offered by the pharmacist at these settings. Contrary to this, pharmacists in Saudi Arabia play promising roles, not only in providing pharmacy services such as dispensing, procurement, and storage, but they are also engaged in dose adjustment, posology, pharmacovigilance, and prescription monitoring to ensure the safe and affordable use of medicines [41]. Patient counselling is of paramount importance for countries such as Pakistan, where self-medication and inappropriate medication-related practices are very common [16,33,42]. However, it is concerning that fundamental counselling services were perceived to be non-existent in community pharmacy settings. These findings are concurrent with the scenario in Northwest Ethiopia and Botswana, as a study reported that counselling was not “so good” as compared to counselling delivered by pharmacists in developed nations [43]. In addition to regulatory shortcomings, fragmented delivery of these emerging patient-oriented pharmacy services at the government, private, and community healthcare levels in Pakistan is attributable to less than optimal academic competence and knowledge of Pakistani pharmacists, and a lack of experience working within novel service provision models. In this regard, a Pakistani study reported that applied drug information, public health and epidemiology, pharmacovigilance, and pharmaco-economics were covered to a lesser degree in the Pharm-D curriculum, and that the majority of pharmacists regarded themselves to be incompetent in these domains [44]. Moreover, being dependent stakeholders [45], working pharmacists in Pakistani hospitals are highly reliant upon administration bodies for licensing and approval. In Pakistan, higher levels of leadership, management, and administrative duties in hospitals are undertaken by medical doctors, and the resulting power imbalance restrains pharmacists in this country from performing in a confident, competent, and assertive manner [20].

Multiple advantages of providing pharmacy services were highlighted, which included benefits related to both patients and the healthcare system. Participants were largely of the opinion that the implementation of pharmacy services in Pakistan could lead to improved quality of life for patients, reduced DRPs, rational use of medicines, and financial gains. Likewise, a study conducted in the UK affirmed that pharmacy services contribute towards sound patient care, positive treatment outcomes, and financial and humanistic benefits [39].

Upon asking the respondents about the obstacles that impede the progression of pharmacy services in Pakistan, they asserted that most of the shortcomings across all sectors and levels within healthcare were due to regulatory processes. The major barriers were suboptimal enforcement of rules and regulations, limited recruitment of pharmacists and consequent insufficient pharmacy workforce, and lack of training opportunities. Similarly, studies conducted in Ethiopia and Malaysia showed that the shortage of pharmacists and insufficient jobs for pharmacists were the major barriers to the implementation of pharmacy services, and these legislative changes were required for proper implementation [46,47]. Even though in Pakistan the National Drug Policy 2003 outlined the goal of implementing fully-fledged pharmacy services and the recruitment of pharmacists in hospitals (one pharmacist per 50 beds), there has been no meaningful improvement in the past two decades. As such, only 0.06 pharmacists are working to cater to the healthcare needs of every 10,000 individuals (6 pharmacists per 1,000,000 population) in Pakistan [20]. Another major factor that impedes the overall progression of pharmacy services is a lack of awareness about the pharmacy profession and pharmacy services among patients and the general public. The issue of lack of awareness about the proactive pharmacist role and service provision has been frequently reported in the international literature, especially from countries with poor literacy rates [48,49,50,51]. The study participants outlined two further perceptions that have been reported in the international literature, including the pharmacists’ practice of renting or leasing out pharmacy license to drug retail outlets without assuring their own presence and negative perceptions from doctors [52,53,54], as barriers to successful implementation of pharmacy services in community and hospital settings, respectively.

To overcome the aforementioned barriers, a number of strategies are proposed from our study. The most important way of helping the situation is to train Pakistani pharmacists well and to equip them with the professional skills required to offer enhanced cognitive services. As evidence of this, a study conducted in Brazil also reported that clinical training and resulting improved knowledge of pharmacists favored the implementation of pharmacy services [30]. Additionally, some participants in our study stated that educating patients about the benefits of pharmacy services and strict enforcement of pharmacy laws could greatly enhance these services. At the hospital level, potential interaction between doctors and pharmacists and the resulting enhanced collaboration was thought to greatly improve the pharmacist’s involvement in patient-centered roles. Similar suggestions have been reported in a US study [55], while a study conducted in Brazil provided evidence that active communication, trust, and respect between pharmacists and physicians promoted improved pharmacy services [32]. However, a Pakistani study also reported that although physicians and nurses consider pharmacists a useful source of information, they regard pharmacist involvement in direct patient care as unpleasant and a menace to hospital harmony [56]. In this regard, mutual goal setting, decision making, and knowledge dissemination between physicians, pharmacists, and patients is essential to advance a team approach to delivering patient care. In order to promote the role of the pharmacist and improve awareness about the benefits of pharmacy services in community settings, pharmacists need to take advantage of their position and should interact directly with the public by ensuring their presence at community pharmacies, rather than renting out pharmacy licenses [19,20]. In doing so, pharmacists will uncover a large window of opportunity associated with community pharmacy service provision. Nevertheless, in this regard, the remuneration model must be revised, along with the establishment of cultures that attract and retain experienced pharmacists [20]. The issue of remuneration [57,58,59] and appropriate pharmacy cultures [60,61,62] within which to deliver services have been dominant discourses in the pharmacy literature in developed nations.

This study contributes to a sparse literature around perceptions of the scope of implementation of pharmacy services in Pakistan. However, there are limitations to this study. Firstly, the study participants were all from a single university in one city in Pakistan. As such, the findings cannot be generalized [29] across the whole of Pakistan. However, similar healthcare policies regarding pharmacy service implementation is common to all of Pakistan. Because the practices of pharmacists are thought to be similar throughout the country, the findings are expected to be similar across Pakistan. Secondly, only pharmacists were included in this study, while the perspectives of policymakers and other members of the healthcare team were not explored. Nevertheless, the views of pharmacists as key stakeholders provide a strong base for future multi-perspective qualitative and quantitative broad surveys to better assess the situation and propose improvements. Finally, by their very nature, pharmacists undertaking postgraduate study may be more engaged with the provision of enhanced cognitive services, and so their views may not reflect the general pharmacist population.

## 5. Conclusions

The participants in this study had an understanding of the terms related to “pharmacy services”. Participants suggested that traditional pharmacy services, such as dispensing and procurement of medicines, were offered across various healthcare settings. Meanwhile, counselling, prescription monitoring, pharmacovigilance, poison control centers, drug utilization review, pharmaco-economics, and pharmaco-epidemiology are suboptimal in Pakistan. The pharmacists considered the reduction of medication-related problems and financial benefits to the healthcare system as possible benefits derived from pharmacy service provision. This study has identified strategies to improve pharmacy services, including campaigns for greater awareness of patients about pharmacy services, proper implementation of the DRAP Act 2012 rules and regulations, better collaboration amongst healthcare professionals, and more robust training opportunities for pharmacists. A number of barriers that have hindered the successful implementation of pharmacy services in Pakistan were identified. These involve various healthcare disciplines in Pakistan, including pharmacists themselves, physicians, and patient-related barriers. Future research should focus on the viewpoint of health policy-makers in Pakistan.

## 6. Impact of Findings on Policy, Practice, and Future Research

Pharmacy services in Pakistan are not fully implemented at all levels of healthcare, including public and private sector hospitals or within community drug retail outlets. The findings of this study are expected to assist policy-makers, especially those within the Pharmacy Services Division of DRAP, to take multifaceted corrective measures to overcome barriers preventing successful implementation of pharmacy services in Pakistan. This study outlines the current scenario of pharmacy services in Pakistan and provides strategies to improve enhanced cognitive pharmacy service provision in this nation. A national survey of pharmacists could verify the generalizability of these findings. It is recommended that initially policy-makers implement these strategies at a pilot scale and later scale this up upon achieving successful results. This study serves as a basis for future in-depth studies to explore the views of the wider healthcare team and policy-makers involved in developing pharmacy services policy in Pakistan.

## Figures and Tables

**Table 1:** Characteristics of the respondents who were interviewed.

Respondent	Gender	Study Specialization	Interview Duration (min)
Pharmacist 1	Female	Pharmaceutical chemistry	27
Pharmacist 2	Male	Pharmaceutical chemistry	29
Pharmacist 3	Female	Pharmaceutics	24
Pharmacist 4	Female	Pharmaceutics	28
Pharmacist 5	Male	Pharmacology	22
Pharmacist 6	Female	Pharmaceutical chemistry	25
Pharmacist 7	Male	Pharmaceutics	27
Pharmacist 8	Male	Pharmacology	24
Pharmacist 9	Female	Pharmaceutics	30
Pharmacist 10	Male	Pharmaceutics	26
Pharmacist 11	Female	Pharmacology	28
Pharmacist 12	Male	Pharmaceutical chemistry	24
Pharmacist 13	Male	Pharmaceutics	30
Mean duration			26.4

**Table 2:** Current scenario of pharmacy services, subthemes, categories, and exemplar quotations.

Theme 1: Current Scenario about Pharmacy Services		
Subthemes	Categories	Quotations
Background knowledge about pharmacy services	Understanding of the term “pharmacy services” Most of the pharmacists were aware of the term “pharmacy services”.	“I am familiar with the term ‘pharmacy services’. This is actually a broad term covering all the services related to the pharmacy and pharmacists. These services are being provided for the welfare of patients (Pharmacist 1)”. “The services provided by the pharmacists in monitoring of prescription, poison control centers, storage, distribution, drug procurement, counselling of patients, dispensing of medicines, drug utilization review, and evaluation for the betterment of patients (Pharmacist 6)”.
	Understanding about the domains within “pharmacy services” Adequate knowledge about the domains within pharmacy services. Limited knowledge about a few domains.	“Yes, as a pharmacist, I have a sound knowledge about dispensing, counselling, selection, distribution, storage, adverse drug reaction, prescription evaluation, and procurement of drugs (Pharmacist 4)”. “Pharmaco-epidemiology, posology, and pharmaco-economics are the least familiar terminologies for the pharmacists with regard to pharmacy services (Pharmacist 2)”.
Pharmacy services being offered	Commonly delivered services Dispensing. Storage, sale, procurement, and distribution.	“Dispensing is the most important role of the pharmacist, which is being offered in Pakistan (Pharmacist 2)”. “Basically, the role of the pharmacist as procurement officer is very important in our health department. Now procurement officers are being hired particularly for this purpose. Drug storage and distribution are also the important roles of pharmacists (Pharmacist 1).”
	Partially delivered services Counselling. Posology and selection of medicines. Prescription monitoring. Pharmacovigilance. Poison control centers. Drug utilization review and evaluation.	“If we talk about pharmacy services then obviously the pharmacists counsel the patients about the medicines according to the prescription. The pharmacists give information about dose, route of administration, precautions, and how to take medicines (Pharmacist 1)”. “Selection and dosage of medicines by the pharmacists are only done at the hospital level. I have seen dose adjustment only at one pharmacy. Some private setups such as Shaukat Khanum, Agha Khan, and Children’s Hospital are providing these services. Other institutes are not performing these services (Pharmacist 8)”. “If the pharmacist monitors the prescription then doctors are not in favor of it. They are egotistic. They do not want their prescriptions changed by pharmacists and they do not want to admit their mistakes. But these services are provided at a few hospitals in Pakistan, such as Shaukat Khanum (Pharmacist 9)”. “Pharmacovigilance is the emerging field in Pakistan. The Drug Regulatory Authority of Pakistan (DRAP) has made a website on it and are trying to improve the adverse drug reaction (ADR) reporting system. The forms are available on the website for reporting any adverse effects. However, this is done at an initial level; only a specified person reports ADRs to the DRAP. I think people do not have knowledge about it (Pharmacist 8).” “There is no such type of poison control center in Pakistan. Some private hospitals are providing these services. They should be available at every teaching hospital and large hospitals (Pharmacist 9).” “Drug utilization evaluation and review are done by the World Health Organization (WHO), not by the government hospitals. These services are also provided at the initial level in Pakistan (pharmacist 12)”.
	Non-existent services Pharmaco-economics and pharmaco-epidemiology.	Pharmaco-economics is only done by M.Phil students, but **is** not implemented in the hospitals (Pharmacist 12).” “Pharmaco-epidemiology is only studied by research students, otherwise there is no such setup in the government sector (pharmacist 11)”.

**Table 3:** Benefits of pharmacy services, subthemes, categories, and exemplar quotations.

Theme 2: Benefits of Pharmacy Services		
Subthemes	Categories	Quotations
To the patients	Improve quality of life for patients. Minimization of drug-related problems. Improve medication adherence. Safe and effective use of medicine.	“These pharmacy services will help to cure the disease and implementation of these services will improve the quality of life of patients (Pharmacist 4)”. “There is a strong need for these services. Medical facilities are not complete without proper implementation of pharmacy services. There are many benefits of pharmacy services, such as better treatment facilities for the community and the proper utilization of drugs. The rate of overdosing, drug interactions, and adverse effects of drugs will be reduced (Pharmacist 3)”. “Patient compliance is one of the important parameters and advantage of these pharmacy services, if implemented (Pharmacist 2)”. “The government needs to establish the pharmacy services in order to provide safe and effective uses of medicines to reduce the adverse drug reactions and mortality rate. These are occurring due to misuse of drugs and errors in the dose selection and administration of drugs (Pharmacist 5)”.
To the healthcare system in Pakistan	Decreased burden on healthcare professionals. Financial savings.	“Obviously there is a need for pharmacists. Because the pharmacist is the expert of medicines. On the other hand, the burden on healthcare professionals should be overcome by the presence of the pharmacists (Pharmacist 8)”. “The implementation of pharmacy services at each level may give financial advantages to our healthcare system by promoting the rational use of medicines and reducing the hospital stays and medicine costs (Pharmacist 11)”.

**Table 4:** Barriers to implementation of pharmacy services, subthemes, categories, and exemplar quotations.

Theme 3: Barriers to Implementation of Pharmacy Services		
Subthemes	Categories	Quotations
Pharmacists	Limited number of pharmacists. Inadequate job opportunities for pharmacists. Lack of training, knowledge, and communication skills. Renting out license of pharmacists.	“The basic barrier is actually the lack of pharmacists in our institutions. We know that ‘majority is authority’. So, we are smaller in number; we have a lot of responsibilities to perform (Pharmacist 1)”. “The employment opportunities for pharmacists are not adequate. Pharmacists are not paid according to their level of qualification. I think this is the main reason (Pharmacist 6)”. “The barriers to implementation of pharmacy services are lack of understanding, lack of communication skills, and lack of training and confidence of pharmacists. Pharmacists need to improve their knowledge about clinical pharmacy. Pharmacists should be capable of performing proper pharmacy services. (Pharmacist 5)”. “One of the important factors is the renting out categories to pharmacies and medical stores. This is against the law and also the basic hurdle in implementation of services (Pharmacist 8)”.
Doctors’ perceptions	Negative perceptions of doctors.	“Negative feedback is always from the doctors. They are not even accepting the role of pharmacists. Doctors consider us their competitors, but actually pharmacists are not their competitors. They are just supporters. And we say that we are actually the life savers because in my point of view, pharmacists play an important role in saving lives of patients (Pharmacist 1)”.
Patients’ awareness of pharmacy	Lack of awareness about pharmacy profession and pharmacy services.	“There is a lack of awareness of patients related to the role of pharmacists, because patients do not rely on the skills of pharmacists. Lack of patient understanding about pharmacy services is also a barrier (Pharmacist 11)”.
Lack of recognition by the Government	Weak enforcement of rules and regulations. No support from the government.	“Policies related to pharmacy services and structure are not implemented. Our Punjab Pharmacy Council is present but not working according to The Pharmacy Act 1967 (Pharmacist 8)”. “There is less support by the Government towards the administration of pharmacy services. Pharmacists cannot work independently (Pharmacist 11)”.

**Table 5:** Strategies to improve pharmacy services, subthemes, categories, and exemplar quotations.

Theme 4: Strategies to Improve Pharmacy Services		
Subthemes	Categories	Quotations
Training opportunities for pharmacists	Provision of proper training and courses. Facilitation of one-year rotation job program to the pharmacists. Improve professional skills of pharmacists.	“Proper training and different courses will facilitate the implementation of the pharmacy services. The pharmacists should be aware of their roles at each level of the healthcare system. In poison control centers, they should have knowledge about all antidotes and treatment protocols. And all of this is possible through proper training and knowledge (Pharmacist 1)”. “First of all, the presence of pharmacists at each level is important. The pharmacists should be provided one-year house jobs for knowledge improvement and competency. This will facilitate implementation of pharmacy services (Pharmacist 8)”. “It is the duty of the pharmacist to improve their practice and professional skills. If they are good in terms of their professional skills, this will be beneficial. The pharmacists should have dedicated their skills towards hospitals and pharmacy services (Pharmacist 2)”.
Make patients more aware	Provide knowledge to patients about pharmacy services. Communication gap between patients and pharmacists.	“The awareness of patients about pharmacy services should also be encouraged. This is the better way to improve these services in Pakistan. Now, most of the people know about these services (Pharmacist 4)”. “Communication gaps between patients and pharmacists can be covered through proper counselling. If we counsel the patients and build up their confidence, then patients will feel the demand of these services. It will help to promote the pharmacy services (Pharmacist 4)”.
Implementation of pharmacy services rules	Enforcement of pharmacy services according to the DRAP Act, 2012.	“I think the regulatory body should strictly hire the pharmacists for 24 hours in hospitals to promote the involvement of pharmacists with patients. It will promote the involvement of pharmacists with patients and ultimately expand the awareness of pharmacy services. I think the regulatory body and Ministry of Health should be at the forefront to incorporate the clinical and pharmaceutical services according to DRAP (Pharmacist 6)”.
Collaboration amongst healthcare providers	Need for better interaction between doctors and pharmacists.	“The interaction between the doctors and pharmacists can be improved by reducing the communication gap between them. The pharmacy services will be better performed in a cooperative environment (Pharmacist 7)”.

## Supplementary Materials

The following are available online at https://www.mdpi.com/1660-4601/17/7/2379/s1, Supplementary File S1: Interview schema.
