# Supplementary material for: Pharmacy Services beyond the Basics: A Qualitative Study to Explore Perspectives of Pharmacists towards Basic and Enhanced Pharmacy Services in Pakistan
Source: Int J Environ Res Public Health. 2020 Mar 31;17(7):2379. doi: 10.3390/ijerph17072379 (PMC7177847; doi:10.3390/ijerph17072379)
Supplement: Supplementary file 1 [file ijerph-17-02379-s001.pdf]

Supplementary File 1

"Interview schema for pharmacists"

Q1. Are you familiar with the term pharmacy services?

- ✓ If yes, please explain
- ✓ Who provides pharmacy services?

Q2. What type of pharmacy services are being offered in Pakistan?

- ✓ Selection, posology, counseling, dispensing, use, administration, prescription monitoring, storage, sale, procurement, distribution pharmacovigilance, pharmacoepidemiology, Pharmacoeconomics, poison control, drug utilization review, drug utilization evaluation

Q3. At which level these services can be or are being provided?

- ✓ Healthcare institutions (pharmacy, medical store, hospital medical institution)
- ✓ Are healthcare institutions providing pharmacy services?

Q4. Do you think that these pharmacy services are according to existing pharmacy rules?

- ✓ If yes, please explain
- ✓ If no, then why?

Q5. In your opinion, is there a need of pharmacy services in Pakistan?

- ✓ If yes, then give reason
- ✓ Patient safety
- ✓ Economic
- ✓ Improve disease management
- ✓ Improve quality of life and health outcomes

Q6. In your opinion, why pharmacy services are not offered in Pakistan?

Q7. How we can implement pharmacy services in Pakistan?

- ✓ Is there anything that should be changed and at what level to make this easier?
